# Emerging Role of Ubiquitin-Specific Protease 19 in Oncogenesis and Cancer Development

**DOI:** 10.3389/fcell.2022.889166

**Published:** 2022-05-12

**Authors:** Fabiana Alejandra Rossi, Mario Rossi

**Affiliations:** Genómica Funcional y Ciencia de Datos, Instituto de Investigaciones en Medicina Traslacional (IIMT), CONICET-Universidad Austral, Buenos Aires, Argentina

**Keywords:** PTMs, ubiquitination, DUBs, USP19, tumorigenesis, metastasis

## Abstract

Ubiquitination and ubiquitin-like post-translational modifications control the activity and stability of different tumor suppressors and oncoproteins. Hence, regulation of this enzymatic cascade offers an appealing scenario for novel antineoplastic targets discovery. Among the different families of enzymes that participate in the conjugation of Ubiquitin, deubiquitinating enzymes (DUBs), responsible for removing ubiquitin or ubiquitin-like peptides from substrate proteins, have attracted increasing attention. In this regard, increasing evidence is accumulating suggesting that the modulation of the catalytic activity of DUBs represents an attractive point of therapeutic intervention in cancer treatment. In particular, different lines of research indicate that USP19, a member of the DUBs, plays a role in the control of tumorigenesis and cancer dissemination. This review aims at summarizing the current knowledge of USP19 wide association with the control of several cellular processes in different neoplasms, which highlights the emerging role of USP19 as a previously unrecognized prognosis factor that possesses both positive and negative regulation activities in tumor biology. These observations indicate that USP19 might represent a novel putative pharmacologic target in oncology and underscores the potential of identifying specific modulators to test in clinical settings.

## Introduction

Following translation, proteins can undergo several posttranslational modifications (PTMs) to modulate their activity, such as phosphorylation, methylation, glycosylation, acetylation, sumoylation and ubiquitination. These modifications represent a very important component in the physiological regulation of different pathways, including protein degradation, DNA repair activity, gene regulation and signal transduction, among others (Millar et al., 2019). Since growth regulatory proteins that drive tumorigenesis are modified by PTMs (Krueger and Srivastava, 2006), understating the mechanisms by which these modifications regulate oncogenic, or tumor suppressive pathways is of great relevance to restrain their effects upon pathological scenarios (Konstantinopoulos et al., 2007).

Moreover, the alteration in the levels and functionality of the components comprising the pathways responsible for the different PTMs, is related to different pathologies, including cancer (Xu et al., 2018; Sharma et al., 2019; Chen et al., 2020; Vellosillo and Minguez, 2021). In particular, ubiquitin-related PTMs are under active study as their dysregulation has been linked with the onset and progression of different oncological disorders (Reinstein and Ciechanover, 2006; Shi and Grossman, 2010).

## Ubiquitination

Ubiquitination is the covalent attachment of ubiquitin (an 8-kDa 76 amino-acid molecule) to target proteins, and it plays crucial roles in the regulation of target proteins activity, stability, subcellular localization and trafficking, and interaction with other proteins (Damgaard, 2021). Therefore, this modification affects a great number of biological processes (Mevissen and Komander, 2017).

**FIGURE 1:**
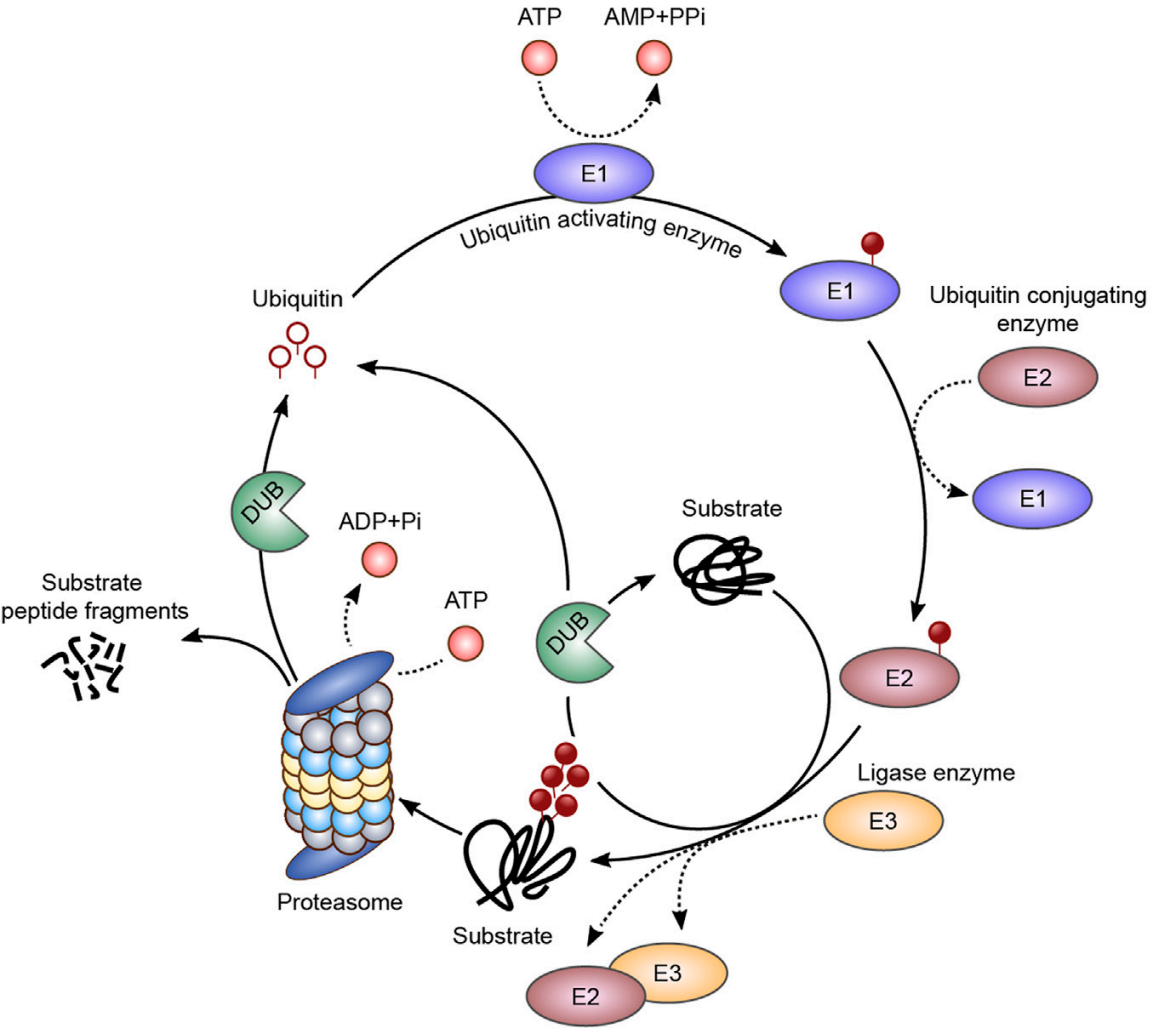
Ubiquitination pathway. The ubiquitin molecule is activated by an E1 ubiquitin activating enzyme, in an ATP-dependent step, and a thioester intermediate is formed (E1-S-ubiquitin). The ubiquitin molecule is then transferred to an E2 conjugating enzyme (E2-S-ubiquitin), and then to the final substrate by an E3 ligase. Ubiquitin bound as monomers or polymers with different topologies are associated with different biological outputs, such as regulation of enzymatic activity, localization, protein-protein interactions, among others. Sequential ubiquitin conjugations form a polyubiquitin chain on the substrate, which can be recognized and degraded by the 26S proteasome. The deubiquitinating enzymes (DUBs) are responsible for the ubiquitin molecules recycling and chain editing.

Protein ubiquitination is a tightly regulated process which involves the activity of two groups of enzymes, namely, E1/E2/E3 ligases and deubiquitinating enzymes (DUBs) (Figure 1).

The attachment of ubiquitination moieties to target proteins is catalyzed by the sequential action of a ubiquitin ATP-dependent activating enzyme (E1), which transfers the ubiquitin molecule to a ubiquitin conjugating enzyme (E2) by trans-thiolation, and by a ubiquitin ligase (E3), which provide substrate specificity to ubiquitin conjugation (Ciechanover, 1994; Hershko and Ciechanover, 1998; Komander and Rape, 2012).

This modification can occur as ubiquitin monomers or polymer chains, and since the ubiquitin molecule contains eight ubiquitination sites (seven internal lysine residues -Lys 6, 11, 27, 29, 33, 48 and 63- and a primary amine at the N-terminus), various types of ubiquitin chains with different length and shape might form (Akutsu et al., 2016; Yau and Rape, 2016; Dwane et al., 2017; Kwon and Ciechanover, 2017; Ohtake and Tsuchiya, 2017).

Furthermore, the ubiquitin molecule is subject to other PTMs such as phosphorylation, acetylation (Ohtake et al., 2015; Wauer et al., 2015; Huguenin-Dezot et al., 2016), and modification with ubiquitin-like proteins such as interferon (IFN)-stimulated gene 15 (ISG15) (Fan et al., 2015) and small ubiquitin-related modifier (SUMO) (Lamoliatte et al., 2013). Therefore, these modifications broaden the ubiquitin code versatility, as they affect not only ubiquitin interactions but also the formation and topology of the polyubiquitin chain.

The nature of the ubiquitin chain determines the outcome of the substrate protein, and different molecular signals are induced in the cell (Ikeda and Dikic, 2008; Sadowski and Sarcevic, 2010), affecting biological processes such as protein stability through proteasome degradation, DNA repair and replication, signal transduction, gene regulation, molecule trafficking and endocytosis, etc. (Hershko and Ciechanover, 1998; Haglund and Dikic, 2005; Komander and Rape, 2012; Yau and Rape, 2016).

The deubiquitinating enzymes are proteases that reverse the modification of proteins by a single ubiquitin or ubiquitin-like protein, and remodel polyubiquitin/ubiquitin-like chains on target proteins. They hydrolyze the isopeptide bond between the ubiquitin and the substrate residue of either the target protein or another ubiquitin molecule (Komander et al., 2009; Komander and Rape, 2012). The human genome encodes nearly 100 DUBs, each with distinct substrate specificities and catalytic properties, which confer high precision upon ubiquitin chains processing (Komander et al., 2009; Mevissen and Komander, 2017). Consequently, individual DUBs likely confer specific actions (Komander et al., 2009; Huang and Dixit, 2016) and pharmacological modulation of their catalytic activity should lead to desired outcomes upon physiological or pharmacological scenarios.

Based on sequence and structural similarities, DUBs have been classified into seven families: Ubiquitin-specific proteases (USPs), Ubiquitin C-terminal hydrolases (UCHs), ovarian tumor proteases (OTUs), Machado-Joseph (Josephin) domain (MJD) proteases, Jab1/MPN domain-associated metallo-iso-peptidases (JAMM/MPM+), Zinc finger UB-specific proteases (ZUP/ZUFSP), and monocyte chemotactic protein-induced proteins (MCPIP). Except for the JAMMs, which are zinc-dependent metalloproteases, the remaining families are cysteine proteases (Reyes-Turcu et al., 2009; Hanpude et al., 2015; Mevissen and Komander, 2017; Kwasna et al., 2018).

## General Properties of USP19

**FIGURE 2:**
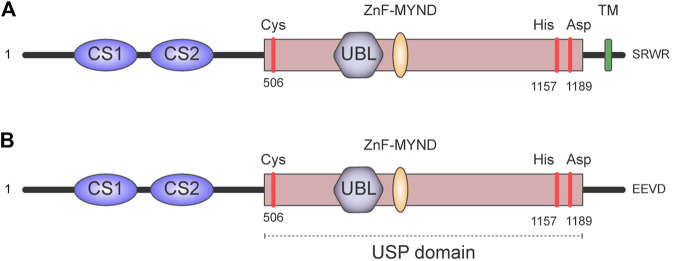
Domain architecture of USP19. It contains two CHORD-SGT1 domains (namely CS1 and CS2) at its N-terminus and a large USP domain with a ubiquitin-like domain (UBL) and myeloid translocation protein 8, Nervy protein, Deaf-1 zinc finger (MYND Zn-finger). The positions of the amino acids Cys, His, and Asp in the catalytic triad are indicated in red. There are multiple USP19 isoforms generated by alternative splicing. In particular, alternative splicing of the last exon generates isoforms with a cytoplasmic localization or isoforms anchored to the endoplasmic reticulum. This schematic depicts: **(A)** the USP19 isoform that contains a transmembrane (TM) domain which anchors USP19 to the endoplasmic reticulum. **(B)** The soluble USP19 isoform has a relatively hydrophilic region and an EEVD motif in the C-terminus instead.

Human ubiquitin-specific protease 19 (USP19) is a modular deubiquitinating enzyme that belongs to the largest family of DUBs, the USPs (Nijman et al., 2005; Reyes-Turcu et al., 2009). This family is characterized by the presence of a highly conserved USP catalytic domain fold (Hu et al., 2002; Hu et al., 2005; Avvakumov et al., 2006; Renatus et al., 2006; Komander et al., 2008), which holds two well-conserved motifs (Cys and His boxes), each containing the critical residues for the enzymatic activity. Moreover, USP19 contains two CHORD-SGT1/P23 domains (namely CS1 and CS2) at its N-terminus, which are relevant for the interaction with other proteins, as well as for the intra-molecular inhibition and regulation of the catalytic core (Xue et al., 2020) (Figure 2).

USP19 presents different isoforms generated by alternative splicing, and the most distinctive feature—structurally and functionally–is that some of them have a cytoplasmic localization, while others have a transmembrane domain that serves as anchorage to the endoplasmic reticulum (Hassink et al., 2009) (Figure 2).

Like other DUBs, USP19 is covalently modified by PTMs such as phosphorylation and ubiquitination, which affect its activity and half-life, respectively (Matsuoka et al., 2007; Velasco et al., 2013).

Functionally, USP19 has mainly been associated with protein quality control and cellular homeostasis (Hassink et al., 2009; Lee et al., 2014; Wiles et al., 2015; He et al., 2016; He et al., 2017), muscle development (Combaret et al., 2005; Sundaram et al., 2009; Wiles et al., 2015), and it has been shown that it controls the half-life of several proteins such as HIF1-α (Altun et al., 2012), BECN1 (Cui et al., 2016), TGFßRI (Zhang et al., 2012), TRAF3 (Gu et al., 2017), HRD1 (Harada et al., 2016), TAK1 (Lei et al., 2019), KPC1 (Lu et al., 2009), c-IAPs one and 2 (Mei et al., 2011), HDAC1/2 (Wu et al., 2017), COROA2 (Lim et al., 2016), LRP6 (Perrody et al., 2016) and MARCH6 (Nakamura et al., 2014), therefore affecting cellular processes relevant in tumorigenesis such as DNA damage repair, apoptosis, the TGF-β Pathway, hypoxia and angiogenesis, immunity, proliferation, ERAD and autophagy.

## The Role of USP19 in Cancer Malignancy

Disrupted regulation of protein ubiquitination is a trigger of cancer, among other diseases. Not surprisingly, alterations in the levels of the ubiquitination cascade components -including the DUBs-have been associated with multiple neoplasms (Shi and Grossman, 2010; Deng et al., 2020; Sun et al., 2020).

In the last couple of years, increasing evidence has begun to demonstrate that USP19 is associated with tumor progression and that it represents a novel prognostic factor for the outcome of several malignant diseases. In particular, it has been shown that USP19 plays both positive and negative roles in the onset and development of diverse neoplasms, in a tissue-specific manner. Consequently, in the following paragraphs, results denoting USP19 relevance in different signaling pathways regulating cell proliferation and cell-cycle progression, as well as tumor growth and metastasis will be presented, therefore unveiling the importance of conducting extensive studies to further the study of USP19’s dual role in tumorigenesis under different molecular scenarios, and to establish its significance as a potential new target for the clinical treatment of cancer.

### USP19 as a DUB Negatively Regulating Tumorigenesis

A couple of recent papers presented results indicating that USP19 negatively affected proliferation and migration in clear cell renal cell (Hu et al., 2020) and serous ovarian carcinomas (Kang et al., 2021).

Hu and others utilized clear cells renal cancer (ccRCC) cell lines *in vitro* and demonstrated that overexpression of USP19 levels negatively affected migration and proliferation, and the opposite occurred upon USP19 silencing. They validated their results using *in vivo* models and observed that USP19 downregulation promoted tumor growth in a xenograft model. Moreover, they conducted *in silico* analyses and observed that USP19 mRNA levels were significantly lower in ccRCC than normal tissues, and that low USP19 expression was associated with disease progression and poor prognostic outcomes in a The Cancer Genome Atlas (TCGA) cohort of patients (Hu et al., 2020). These results were consistent with a previous work by Liu and collaborators, who performed an *in silico* analysis and observed that isoform uc003cvz.3, which is mainly localized in the cytoplasm, serves as an indicator of poor outcome in patients with advanced stage ccRCC (Liu et al., 2013).

Similarly, Kang et al. applied a machine learning model on RNA-sequencing data from 51 patients who received conventional therapies for high-grade serous ovarian carcinoma (HGSC) and identified USP19 and RPL23 as candidate prognostic markers. Specifically, they showed that patients with lower USP19 or higher RPL23 mRNA levels had worse prognoses and they validated their model using publicly available data from the TCGA (Kang et al., 2021). They also observed that USP19 levels positively correlated with TOP3B and XRN2, which regulate genome instability (Kang et al., 2021). Based on this observation, and considering that USP19 interacts with and deubiquitinates HDAC1/2 in order to regulate DNA damage repair and chromosomal stability (Wu et al., 2017) and that both ccRCC and HGSC are characterized by high genomic instability, it is plausible that USP19-mediated deubiquitination of key regulators associated with DSB repair or genome instability might be responsible for the worse prognosis observed in ccRCC and HGSC patients with low USP19 levels (Kang et al., 2021).

In addition, Shahriyari L and collaborators (Shahriyari et al., 2019) described the existence of a correlation between the expression of USP19, RBM15B and the tumor suppressor gene BAP1 (BRCA1 associated protein-1) in different type of cancers. All three genes are in proximity of the 3p21 tumor suppressor region, which is commonly altered in many cancers, suggesting that USP19 could play a functional role in BAP1 molecular mechanism of action or its alteration could be a byproduct of chromosomal rearrangement affecting other genes. Although further characterization is required, this observation highlights the potential of USP19 as a putative prognostic biomarker in different cancers.

### USP19 Positively Regulates Tumor Growth and Metastasis

Opposite to the role of USP19 as a tumor suppressor, recent work has also established that antagonism of USP19 expression conferred a prominent antiproliferative and antitumorigenic response in diverse neoplasms: Ewing sarcoma, gastric, breast and colorectal cancers (Gierisch et al., 2019; Dong et al., 2020; Rossi et al., 2021; Zhu et al., 2021), suggesting pro-tumorigenic roles in these tissues.

Ewing sarcoma is the second most common pediatric bone and soft tissue tumor, which is characterized by the presence of a chimeric oncoprotein, EWS-FLI1, due to a genetic translocation between chromosomes 22 and 11 (Desmaze et al., 1997). Gierisch and collaborators demonstrated that this protein, which maintains tumor cells survival, is regulated by USP19 in a post translational manner, and dependent on its catalytic activity (Gierisch et al., 2019). Downregulation of USP19 levels resulted in a reduction of EWS-FLI1 levels, hence decreasing tumor cells growth and colony formation capability, whereas the opposite occurred upon USP19 (TM isoform) overexpression. Using *in vivo* experiments, the authors demonstrated that tumor growth was delayed when USP19 levels were reduced.

On the other hand, Dong and others analyzed USP19 relevance in gastric cancer (Dong et al., 2020). Their results revealed that USP19 TM isoform overexpression enhanced cell proliferation and exhibited anti-apoptotic properties, as well as it increased cells migration and spreading capabilities *in vitro*; the opposite was observed upon USP19 silencing (multiple isoforms). Furthermore, they showed that increased USP19 (TM isoform) levels enhanced MMP2/MMP9 protein expression and enzyme activity, and that genetic alteration of USP19 levels affected tumorigenesis using *in vivo* models. Finally, using a cohort of 212 gastric cancer patients, the authors observed that USP19 expression was significantly increased in gastric cancer tissues, compared to normal gastric tissues, and the high level of USP19 expression was positively correlated with a poorer prognosis.

Similarly, our group analyzed USP19 clinical significance in breast cancer (Rossi et al., 2021). We demonstrated that USP19 positively regulates breast tumor cells migration and invasion *in vitro*, and that genetic silencing reduces cells motility, whereas its overexpression increases migratory and invasive capabilities—dependent on USP19’s catalytic activity and ER localization. Our results also indicated that USP19 does not affect breast cancer cells proliferation in two dimensions, in concordance with Lu and collaborators (Lu et al., 2011), but significantly modulates proliferation and invasion if cells are grown embedded in extracellular matrix proteins and basement membrane proteins. *In vivo* experiments showed that USP19 silencing reduces tumorigenicity and delays tumor onset and growth, and the opposite was observed upon wild type USP19 overexpression (but not when overexpressing a catalytically dead mutant, or a cytoplasmic version of USP19). Using experimental metastasis assays, we verified that USP19 silencing reduces cells’ ability to engraft in secondary tissues, and using *in silico* approaches and TCGA data, we demonstrated that the Wnt pathway is activated in patient samples expressing high levels of USP19. In concordance with these results, we observed a positive correlation between USP19 and LRP6 levels (a Wnt pathway coreceptor). Functional analysis on USP19 overexpressing cells indicated that LRP6 silencing reverted migratory and invasive phenotypes, possibly as a downstream USP19 effector. Finally, we conducted a retrospective analysis on early breast cancer patients which revealed that USP19 expression levels correlated with poor outcome and reduced distant metastasis free survival, hence serving as a prognostic factor in early breast cancer patients.

Lastly, a very recent publication by Zhu and collaborators studied USP19 pertinence in colorectal carcinogenesis (Zhu et al., 2021). Their work showed that ERK2 signaling is responsible for lipid synthesis mediated by cytoplasmic-localized malic enzyme 1 (ME1) phosphorylation, which is overexpressed in a variety of cancers (including colorectal cancer). USP19-mediated ME1 stabilization is enhanced by phosphorylation, generating oncogenic phenotypes, and either USP19 deletion or a point mutation in ME1 protein that prevents ubiquitination, represses colorectal carcinogenesis. Of note, USP19 catalytic activity is necessary to ensure ME1 stabilization. Finally, the authors showed that the USP19-ME1 signaling axis is dysregulated in human colorectal cancer samples, and that USP19 is upregulated during colorectal carcinogenesis pathogenesis and spontaneous tumor development.

Supplementary Table S1 summarizes USP19 relevance in different cancers, and whether is catalytic activity or specific isoform is important in each type of neoplasm.

## Concluding Remarks

Various studies have linked USP19 to different cancers, and either its overexpression or silencing may dysregulate the function of several proteins with oncogenic or tumor-suppressive properties, which in the long run may impact on the onset and development of tumors. Since USP19 has different isoforms, and divergent effects have been observed in different cancers, it is plausible to assume that this difference could be explained by the effect these isoforms exert on differing substrates. Moreover, USP19 is a fundamental deubiquitinase with pivotal roles in several cellular processes related to tumorigenesis, including DNA damage repair (Wu et al., 2017), apoptosis (Mei et al., 2011), the TGF-β Pathway (Zhang et al., 2012), hypoxia and angiogenesis (Altun et al., 2012; Boscaro et al., 2020), immunity (Cui et al., 2016; Jin et al., 2016; Gu et al., 2017; Lei et al., 2019; Wu et al., 2019; Liu et al., 2021), proliferation (Lu et al., 2009), ERAD (Hassink et al., 2009) and autophagy (Cui et al., 2016). Given its versatility, USP19’s role on tumorigenesis and metastasis might also be determined by a combinatorial effect on diverse signaling pathways rather than a specific substrate. In this respect, more studies should be performed to analyze the association of USP19 with cancer-related signaling pathways and putative targets, regulatory mechanisms affecting its expression and to search for molecular alterations shared by tumors across different tissues and new targets to better understand how USP19 is affecting cell survival and cellular homeostasis.

Taken together, the findings described here implicate USP19 as a previously unrecognized target for the development of novel therapeutic alternatives for cancer treatments.
